# A human antibody epitope map of the malaria vaccine antigen Pfs25

**DOI:** 10.1038/s41541-023-00712-z

**Published:** 2023-08-04

**Authors:** Niharika Shukla, Wai Kwan Tang, Camila H. Coelho, Carole A. Long, Sara A. Healy, Issaka Sagara, Kazutoyo Miura, Patrick E. Duffy, Niraj H. Tolia

**Affiliations:** 1 Host-Pathogen Interactions and Structural Vaccinology Section, Laboratory of Malaria Immunology and Vaccinology, National Institute of Allergy and Infectious Diseases, National Institutes of Health, Bethesda, USA; 2 Pathogenesis and Immunity Section, Laboratory of Malaria Immunology and Vaccinology, National Institute of Allergy and Infectious Diseases, National Institutes of Health, Bethesda, USA; 3 Laboratory of Malaria and Vector Research, National Institute of Allergy and Infectious Diseases, National Institutes of Health, Rockville, MD USA; 4 Vaccine Development Unit, Laboratory of Malaria Immunology and Vaccinology, National Institute of Allergy and Infectious Diseases, National Institutes of Health, Bethesda, USA; 5 Malaria Research and Training Center, University of Sciences, Techniques, and Technology, Bamako, Mali; 6 Present Address: Center for Vaccine Research and Pandemic Preparedness (C-VARPP), Department of Microbiology, Icahn School of Medicine at Mount Sinai, New York, NY USA

**Keywords:** Antibodies, Protein vaccines, Clinical microbiology, Translational research

## Abstract

Pfs25 is a leading antigen for a malaria transmission-blocking vaccine and shows moderate transmission-blocking activity and induction of rapidly decreasing antibody titers in clinical trials. A comprehensive definition of all transmission-reducing epitopes of Pfs25 will inform structure-guided design to enhance Pfs25-based vaccines, leading to potent transmission-blocking activity. Here, we compiled a detailed human antibody epitope map comprising epitope binning data and structures of multiple human monoclonal antibodies, including three new crystal structures of Pfs25 in complex with transmission-reducing antibodies from Malian volunteers immunized with Pfs25 conjugated to EPA and adjuvanted with AS01. These structures revealed additional epitopes in Pfs25 capable of reducing transmission and expanded this characterization to malaria-exposed humans. This work informs immunogen design to focus the antibody response to transmission-reducing epitopes of Pfs25, enabling development of more potent transmission-blocking vaccines for malaria.

## Introduction

Malaria continues to represent a profound global health burden, causing 241 million cases and 627,000 deaths in 2020^1^. The most developed vaccine to prevent malaria infection, RTS,S/AS01, is intended to prevent infection of the human host by targeting the pre-erythrocytic stage of the *Plasmodium falciparum* parasite^2,3^. The *Plasmodium* parasite has a complex life cycle, including stages within the human and the female *Anopheles* mosquito, and vaccine approaches targeting additional stages of the parasite life cycle may complement a pre-erythrocytic stage approach^4,5^. Transmission-blocking vaccines (TBV) represent a promising avenue for malaria intervention by targeting *Plasmodium* sexual stages to prevent parasite development within the mosquito vector, thereby reducing infectivity and transmission within a community^6,7^.

Pfs25, a leading TBV candidate, is a glycosylphosphatidylinositol-anchored protein consisting of four EGF-like domains^8^. Pfs25 is expressed on the surface of zygotes and ookinetes and plays a role in ookinete survival in the mosquito midgut, traversal through the midgut epithelium, and oocyst maturation^9–11^. Though Pfs25 is not a target of naturally acquired immunity in humans, mouse antibodies elicited by Pfs25 immunizations formulated in various adjuvants potently inhibit oocyst development and provide a basis for its development as a TBV^12–16^. Pfs25 is also highly conserved among diverse *P. falciparum* strains with limited polymorphic variation, suggesting immunization with Pfs25 may lead to strain-transcending immunity^17–21^.

Pfs25 has undergone extensive clinical development to assess safety and efficacy in malaria-naïve and malaria-exposed populations. This includes immunogenicity-enhancing approaches through virus-like particle (VLP) display, nanoparticle display, conjugation to *P. aeruginosa* Exoprotein A (EPA) and formulation in diverse adjuvants^22–25^. Despite these efforts, Pfs25 immunization in humans elicits only modest transmission-reducing activity (TRA) and rapidly declining antibody titers^22–25^. Structural characterization of epitopes of several malaria antigens from all stages of the life cycle^26–43^ has provided crucial information about the structural basis of neutralization and guided immunogen design. Identification of transmission-reducing epitopes on Pfs25 may inform structure-based design of next-generation Pfs25 immunogens to focus the immune response towards these transmission-reducing epitopes^6,44^.

Four distinct immunogenic sites on Pfs25 termed Sites 1, 2, 3, and the bridging site have previously been characterized using antibodies derived from Kymice^TM^ and malaria-naïve humans vaccinated with Pfs25-VLP^38,39^. These findings identify Site 1, Site 3, and the bridging site of the antigen as targets of potent transmission-reducing antibodies, and Site 2 as a target of weak transmission-reducing antibodies^38,39^. However, there are still regions of Pfs25 that are structurally uncharacterized with respect to transmission-blocking activity, and it remains unclear whether immunization in a malaria-exposed population will elicit a pattern of immune recognition distinct from that seen in malaria-naïve individuals.

Here, we defined the structural and biophysical characteristics of human transmission-reducing antibodies elicited through Pfs25-EPA/AS01 immunization in malaria-exposed populations (ClinicalTrials.gov NCT02942277). Of fifteen anti-Pfs25 antibodies isolated from four participants, we further characterized five human monoclonal antibodies (hmAbs) which demonstrated highest transmission-reducing activity. We demonstrated that these hmAbs recognize additional epitopes based on epitope binning data, and we determined crystal structures of three representative antibodies in complex with Pfs25. These findings define additional transmission-reducing epitopes and provide a detailed human antibody epitope map of Pfs25.

## Results

### Transmission-reducing hmAbs recognize diverse epitopes on Pfs25

**Fig. 1:**
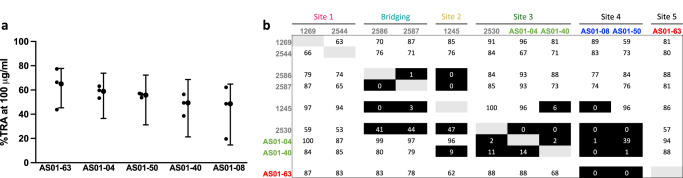
Functional activity and epitope bins of Pfs25 mAbs. **a** Transmission-reducing activity (TRA) of hmAbs elicited by immunization with Pfs25-EPA/AS01. Percent TRA is defined as reduction in oocyst count in the midgut of *Anopheles* mosquitos as measured in Standard Membrane Feeding Assay (SMFA) using an antibody concentration of 100 µg/ml. Individual circles represent data from three biological replicates while the closed circles with error bars represent the best estimated percent TRA and the associated 95% confidence interval calculated as described previously in ref. ^57^. **b** Epitope binning results of human anti-Pfs25 mAbs against reference mAbs indicated by grey text. Saturating mAbs are listed along the left column while competing mAbs are listed along the top of the panel. AS01-08 and AS01-50 were observed to have high dissociation rates from the Pfs25-coated sensor during the baseline and were excluded from the experiment as saturating mAbs but were still suitable for use as competing mAbs. Values indicate percentage of binding of competing mAbs to Pfs25 relative to maximum binding in the absence of competing mAbs. Values less than 50 were assigned as competing mAbs and shaded in black while values greater than 50 were assigned as noncompeting mAbs.

Fifteen antibodies isolated from four Malian individuals vaccinated with Pfs25-EPA/AS01 (NCT02942277) that showed positive interaction with Pfs25 from enzyme-linked immunoassay (ELISA) were named with the prefix “AS01” and a number (Supplementary Table 1). The five hmAbs with the greatest transmission-reducing activity were selected for further characterization (Fig. 1a and Supplementary Fig. 1). To evaluate where these antibodies bound, we performed epitope binning experiments using biolayer interferometry (BLI) with single chain variable fragments (scFvs) of each antibody and a Pfs25 construct consisting of the four extracellular EGF-like domains (Supplementary Fig. 2). Pfs25 was immobilized on a biosensor and then incubated with a saturating mAb to allow for association. The biosensor was then dipped into wells containing a competing mAb while binding was measured to determine whether the saturating and competing mAbs recognized competing or non-competing epitopes. Four immunogenic sites on Pfs25 have been previously characterized using Kymice^TM^ as well as human antibodies induced from Pfs25-VLP immunization (Supplementary Fig. 3)^38,39^. The previously characterized mAbs which recognize Site 1 (mAbs 1269, 2544), Bridging Epitope (mAbs 2586, 2587), Site 2 (mAb 1245), and Site 3 (mAb 2530) were used as references in these competitive binning experiments.

Epitope binning results indicated that the five hmAbs from Pfs25-EPA/AS01 immunization fell into three epitope bins (Fig. 1b). Notably, AS01-63 in Site 5 appeared to recognize a distinct epitope as it did not compete with any previously characterized mAbs. AS01-50 and AS01-08 in Site 4 competed with both Site 3 and Site 5, indicating they may recognize an overlapping epitope between these two sites. AS01-04 and AS01-40 show a similar binding pattern as mAb 2530 in Site 3. None of the five AS01 hmAbs competed with the Site 1 and Bridging Epitope.

### Crystal structures reveal additional epitopes on Pfs25

**Fig. 2:**
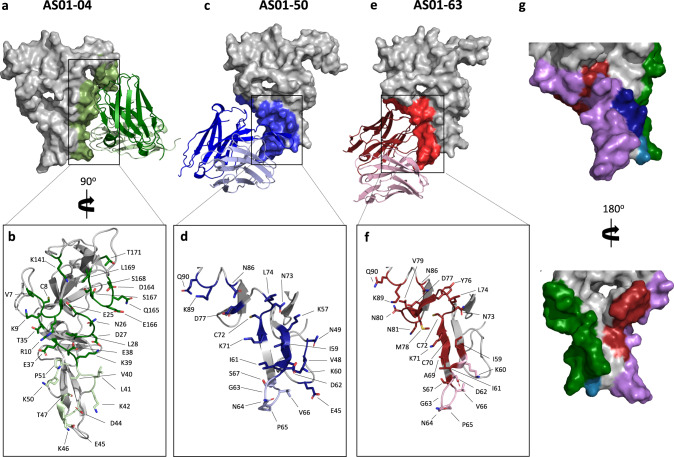
Structures of Pfs25-antibody complexes. **a** Crystal structure of Pfs25 in complex with AS01-04 scFv. Pfs25 depicted as surface and AS01-04 depicted as cartoon with heavy chain in dark green and light chain in pale green. AS01-04 epitope indicated as green surface on Pfs25. **b** Detailed view of AS01-04 epitope. Pfs25 residues which interact with AS01-04 heavy chain indicated in dark green, and Pfs25 residues which interact with AS01-04 light chain indicated in pale green. **c** Crystal structure of Pfs25 in complex with AS01-50 scFv. Pfs25 depicted as surface and AS01-50 depicted as cartoon with heavy chain in dark blue and light chain in pale blue. AS01-50 epitope indicated as blue surface on Pfs25. **d** Detailed view of AS01-50 epitope. Pfs25 residues which interact with AS01-50 heavy chain indicated in dark blue, and Pfs25 residues which interact with AS01-50 light chain indicated in pale blue. **e** Crystal structure of Pfs25 in complex with AS01-63 scFv. Pfs25 depicted as surface and AS01-63 depicted as cartoon with heavy chain in dark red and light chain in pale red. AS01-63 epitope indicated as red surface on Pfs25. **f** Detailed view of AS01-63 epitope. Pfs25 residues which interact with AS01-63 heavy chain indicated in dark red, and Pfs25 residues which interact with AS01-63 light chain indicated in pale red. **g** Comparison of AS01-04, AS01-50 and AS01-63 epitopes. Pfs25 residues which interact with AS01-04 alone are indicated in green, residues which interact with AS01-50 alone are indicated in blue, residues which interact with AS01-63 alone are indicated in red, residues which are within both AS01-04 and AS01-50 epitopes are indicated in teal, and residues which lie within both AS01-50 and AS01-63 epitopes are indicated in purple.

X-ray crystallography was used to solve the structures of representative antibodies from Sites 3 (AS01-04), 4 (AS01-50), and 5 (AS01-63) in complex with Pfs25 (Supplementary Table 2, Supplementary Fig. 4). The crystal structure of Pfs25 in complex with AS01-04 scFv was solved at a resolution of 2.3 Å, revealed an epitope that overlapped with Site 3 in EGF2, as predicted by the epitope binning results, but also extended along previously uncharacterized surfaces of EGF4, near the C-terminus (Fig. 2a, b, Supplementary Fig. 5a). All complementarity-determining regions (CDRs) except for light chain CDR 2 of AS01-04 contacted Pfs25, burying a surface area of 1140 Å^2^. A 805 Å^2^ was contributed by the heavy chain while 335 Å^2^ was contributed by the light chain. The heavy chain formed ten hydrogen bonds and five salt-bridges while one hydrogen bond was formed by the light chain. The complex was also stabilized by extensive hydrophobic interactions (Supplementary Table 3).

The crystal structure of Pfs25 in complex with AS01-50 scFv was solved at a resolution of 2.3 Å (Fig. 2c, d, Supplementary Table 2). AS01-50 recognized a previously uncharacterized conformational epitope within EGF2 and EGF3 of Pfs25 (Supplementary Fig. 5b). This epitope lies on the opposite face of EGF2 relative to the epitopes of the Site 3 hmAbs, 2530 and AS01-04. The close proximity of the two epitopes suggests that the competition of AS01-50 with 2530 and AS01-04 in epitope binning is due to steric hindrance preventing their simultaneous binding, rather than overlapping epitopes (Fig. 2g). All CDRs except heavy chain CDR2 of AS01-50 were in contact with Pfs25, burying a surface area of 912 Å^2^. The majority of this buried surface area (BSA) was contributed by the heavy chain (645 Å^2^) compared to the light chain (267 Å^2^). Notably, the buried surface area of HCDR3 alone (506 Å^2^) contributed to the majority of the total BSA of the antibody. The heavy chain formed six hydrogen bonds and one salt bridge, while the light chain formed five hydrogen bonds. The complex was also stabilized by extensive hydrophobic interactions (Supplementary Table 4).

The crystal structure of Pfs25 in complex with AS01-63 scFv was solved to a resolution of 2.1 Å. AS01-63 recognized a conformational epitope within EGF2 and EGF3 of Pfs25 (Fig. 2e, f, Supplementary Fig. 5c). All six CDRs of AS01-63 contacted Pfs25, burying a surface area of 765 Å^2^. The majority of this BSA was contributed by the heavy chain (594 Å^2^), while the light chain buried 171 Å^2^. The heavy chain formed eight hydrogen bonds while the light chain formed seven hydrogen bonds and four salt bridges. The complex was also stabilized by extensive hydrophobic interactions (Supplementary Table 5). Consistent with the epitope binning results, the epitope of AS01-63 overlapped considerably with that of AS01-50 (Fig. 2g). However, the epitope of AS01-63 extends further into EGF3 of Pfs25, overlapping partially with Site 1 of Pfs25.

### Pfs25 mAbs showed a broad range of binding affinities and kinetics

**Fig. 3:**
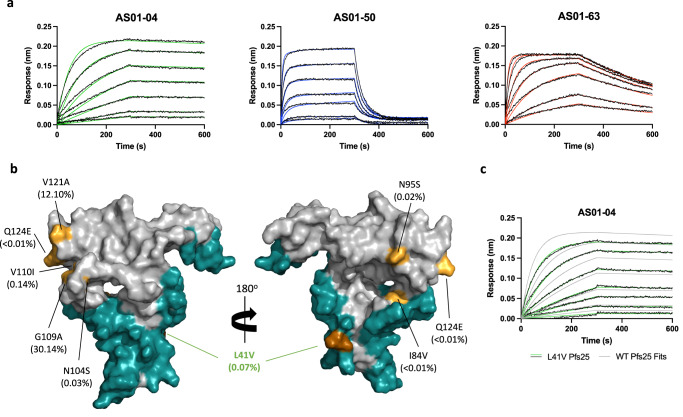
Binding kinetics and polymorphism analysis of Pfs25 mAbs. **a** Representative binding curves of biolayer interferometry experiments using Pfs25 and IgGs. Curves represent two-fold dilutions of Pfs25 concentration. Colored lines indicate best fits of data to 1:1 binding model. **b** Surface representation of Pfs25 with epitopes in teal, polymorphic residues in pale orange, and polymorphisms overlapping with epitopes in dark orange. Polymorphic residues are labelled with frequency according to database of 3488 sequences from MalariaGen indicated in parentheses. **c** Representative binding curves of biolayer interferometry experiments using AS01-04 IgG with Pfs25 L41V. Curves represent two-fold dilutions of Pfs25 concentration. The fitted curves of wildtype Pfs25 from Fig. 4a was overlaid (in gray).

Binding kinetics experiments were conducted for the three structurally characterized Pfs25 antibodies using biolayer interferometry (Fig. 3a, Table 1, Supplementary Table 6). Binding kinetics and affinity were determined for the IgGs of these mAbs to characterize the most physiologically relevant form of the antibody. These antibodies displayed a broad range of binding affinities and kinetics with a greater than 100-fold difference in binding affinity K_D_ from 0.37 nM for AS01-04 to 68.60 nM for AS01-50 (Fig. 3a, Table 1, Supplementary Table 6). This dramatic difference in binding affinity was driven mostly by changes in the disassociation rates (k_dis_), which ranged from 0.09 × 10^−3^ s^−1^ for AS01-04 to 20.49 × 10^−3^ s^−1^ for AS01-50, while the association rates of all three antibodies were similar (Table 1, Supplementary Table 6). This difference in k_dis_ and binding affinity was likely due to differences in the binding interfaces between each complex, as AS01-04 had the largest BSA of 1,140 Å^2^ and an extensive network of 11 hydrogen bonds and 5 salt bridges while AS01-50 had a BSA of 912 Å^2^ and a network of only 11 hydrogen bonds and one salt bridge (Supplementary Tables 3–5).

**Table 1:** Binding affinity (K_D_), association rate (k_a_), and dissociation rate (k_dis_) of hmAbs.

hmAb	Pfs25	K_D_ (x 10^−9^ ± SEM M)	k_a_ (x 10^5^ ± SEM M^−1^s^−1^)	k_dis_ (x 10^−3^ ± SEM s^−1^)
AS01-04	WT	0.37 ± 0.06	2.51 ± 0.10	0.09 ± 0.02
AS01-04	L41V	0.65 ± 0.19	2.11 ± 0.13	0.13 ± 0.03
AS01-50	WT	68.60 ± 4.76	2.84 ± 0.22	20.49 ± 0.55
AS01-63	WT	8.71 ± 1.05	2.28 ± 0.31	1.92 ± 0.04

### Recognition of Pfs25 epitopes is not impacted by common polymorphisms

Analysis of 3488 sequences from MalariaGen^45^ demonstrated that nearly all observed polymorphic residues in Pfs25 lie outside the epitopes identified in this study (Fig. 3b, Supplementary Table 7). The two most common polymorphisms, G109A and V121A, which are present in 30.1% and 12.0% of isolates, were in Site 1 of Pfs25 and lie distant from all the newly characterized epitopes. These findings suggest antibodies elicited by Pfs25-EPA immunization in malaria-exposed populations target broadly conserved epitopes across diverse strains of *P. falciparum*. Only L41V, a polymorphism with a low frequency of 0.07%, was found within the epitope of transmission-reducing mAb, AS01-04. However, the binding affinity of AS01-04 to this polymorphic variant of Pfs25 was not affected compared to wildtype Pfs25 (Fig. 3c, Table 1 and Supplementary Table 6).

### Sequence and germline analysis of Pfs25 mAbs

All Pfs25 antibodies represent distinct pairings of heavy and light chain variable genes (Supplementary Table 8). Using IgBlast, the sequences of each antibody were compared to the germline V, D, and J sequences (Supplementary Fig. 6). AS01-04 underwent 21 amino acid changes from the germline sequence, AS01-50 underwent 9 amino acid changes, and AS01-63 underwent 19 amino acid changes. All antibodies also demonstrated insertions in the heavy chain CDR3, with AS01-50 undergoing the largest insertion of 10 residues. This insertion corresponds to the major binding interface of AS01-50 with Pfs25. AS01-04 and AS01-63 had smaller insertions of 5 amino acids in heavy chain CDR3. Insertions in light chain CDR3 were less frequent, with only AS01-50 undergoing an insertion of one amino acid.

### Epitope map of Pfs25

**Fig. 4:**
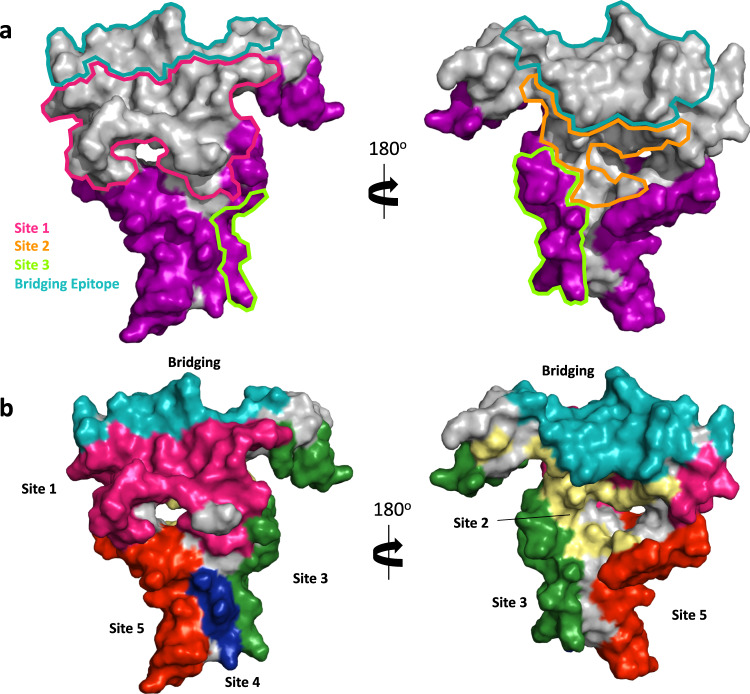
Epitope map of Pfs25. **a** Surface representation of Pfs25. Previously characterized epitopes are outlined, while epitopes of AS01-04, AS01-50, and AS01-63 are shaded in purple. **b** Pfs25 surface highlighted by all new and previously identified immunogenic sites^38,39^.

The epitopes characterized in the crystal structures of AS01-04, AS01-50, and AS01-63 highlighted additional transmission-reducing surfaces on Pfs25. They overlapped considerably with Site 3, but notably do not overlap considerably with Sites 1, 2, or the bridging site (Fig. 4a). Together, these epitopes contribute to a detailed epitope map of Pfs25 and described Site 4 and Site 5 as additional targets of moderate transmission-reducing antibodies (Fig. 4b, Table 2).

**Table 2:** Origin, transmission-reducing activity as measured in Standard Membrane Feeding Assay (SMFA), and binding affinity of Pfs25 mAbs from each site^38,39^.

Name	Epitope	Origin	TRA at 10 μg/ml	TRA at 100 μg/ml	TRA at 375 μg/ml	IC80 (μg/ml)	K_D_ (nM)
**2544**	Site 1	Human	-	100	-	16	4.6 ± 1.2
**1269**	Site 1	Kymouse	−24.4	-	100	63	3.7 ± 0.3
**1262**	Site 1	Kymouse	−25.1	-	100	-	12.0 ± 0.4
**1276**	Site 1	Kymouse	−20.8	-	97.7	-	16.0 ± 2.5
**1190**	Site 1	Kymouse	-	-	54.3	-	6.7 ± 0.7
**2586**	Bridging	Human	-	92.2	-	96	2.5 ± 0.2
**2587**	Bridging	Human	-	84.5	-	-	4.6 ± 2.5
**1245**	Site 2	Kymouse	−39.5	-	93.2	263	31.0 ± 5.6
**1260**	Site 2	Kymouse	−50.2	-	99.2	-	41.0 ± 9.8
**2530**	Site 3	Human	-	88.3	-	65	19.2 ± 1.9
**AS01-04**	Site 3	Human	-	58.7	-	-	0.37 ± 0.06
**AS01-50**	Site 4	Human	-	55.8	-	-	68.60 ± 4.76
**AS01-63**	Site 5	Human	-	62.5	-	-	8.71 ± 1.05

## Discussion

Strategies to enhance immune responses to vaccination in malaria-exposed populations must be explored to further develop Pfs25 as a transmission-blocking vaccine. One such approach is through identification of transmission-reducing epitopes to inform structure-based vaccine design and generate a focused potent immune response. This study represents the first structural and biophysical characterization of human transmission-reducing antibodies raised by immunization with Pfs25 in a malaria-exposed population.

Past work in Kymice^TM^ and malaria-naïve humans has identified four immunogenic sites (Site 1, Site 2, Site 3 and Bridging Epitope Site), with Site 1 as the target of the most potent transmission-blocking antibodies and the majority of previously characterized antibodies^38,39^. None of the antibodies reported in the present study compete with Site 1 antibodies 1269 and 2544. Rather, our findings revealed additional epitopes that create Site 4 and Site 5 as targets of transmission-reducing antibodies. This work contributes to the definition of a detailed Pfs25 epitope map, suggesting the majority of the surface of Pfs25 can be targeted by transmission-reducing antibodies of diverse potency (Fig. 4, Table 2). Detailed antibody epitope maps have facilitated the design of immunogens that enhance the functional immune response^46–48^ and the epitope map presented here will facilitate similar efforts for Pfs25.

AS01-04 overlapped considerably with the previously reported antibody 2530 in Site 3, and its epitope also extended towards the C-terminus region of EGF4, and revealed a surface that has not previously been shown to be the target of transmission-reducing antibodies in Kymice^TM^ or humans^38,39^. Past studies characterized Kymice^TM^ and human^38,39^ antibodies generated after immunization with a virus-like particle consisting of Pfs25 fused to the Alfalfa Mosaic Virus Coat Protein at its C-terminus, potentially limiting its availability for antibody generation^38,39^. Antibodies in the present study were elicited in response to an AS01-adjuvanted vaccination with a Pfs25 construct conjugated to EPA, which may allow greater access to the C-terminus of Pfs25 for generation of transmission-reducing antibodies^49^. Notably, our crystal structures revealed that the C-terminus region of Pfs25 is flexible, and when in complex with AS01-04, adopts a conformation that brings it closer to EGF1. Immunogen design to stabilize this conformation of Pfs25 may elicit an immune response to better target conserved regions and functional conformations of the antigen and demonstrate greater strain-transcending activity.

The epitopes of antibodies isolated from Malian individuals vaccinated with Pfs25 appear distinct from epitopes identified through prior studies. It is notable that none of the antibodies in the present study were found to bind entirely within Site 1, the antigenic site shown to be the target of the majority of reported antibodies^38,39^. It is possible that this lack of response to Site 1 is due to preferential conjugation of EPA to Site 1, masking its surface from immune recognition; or the effect of prior exposure to malaria infection. Alternately, malaria-exposed populations have been shown to exhibit differences in immune response to vaccination and controlled human malaria infection compared to malaria-naïve individuals. For example, malaria-specific non-AMA1 antibodies found in malaria-exposed populations can interfere with the functional activity of AMA1-specific antibodies^50^. It is plausible that differences in germ line sequences and population genetics may contribute to observed differences^51^. Finally, dampening of the B cell response in malaria exposed individuals has been reported^52,53^. Though Pfs25 is not a target of naturally acquired immunity, it is possible that prior malaria exposure may also explain this pattern of antibody recognition of the surface of Pfs25 that is distinct from what has been observed in malaria-naïve individuals and preclinical trials. The mechanisms behind this altered epitope specificity are unclear and should be explored in future population-based studies particularly as monoclonal antibody isolation and characterization may bias the types of antibodies identified.

Overcoming sequence diversity across *Plasmodium* strains is a major challenge to malaria vaccine development^54^. The majority of Pfs25 polymorphisms lie within Site 1, while Site 4 and Site 5 are unaffected by common polymorphisms. Furthermore, the single polymorphism in Site 3 does not affect the affinity of AS01-04. These findings suggest that focusing the immune response to Sites 3, 4, and 5 may elicit broadly transmission-reducing, strain-transcending protection against malaria. Collectively, these findings provide a basis for structure-based design of next-generation Pfs25 immunogens to highlight surfaces of the antigen that may be relevant targets of protection in a malaria-exposed population.

## Methods

### Human ethics statement

This study was approved by the ethics review boards from the Faculté de Médecine de Pharmacie et d’Odonto Stomatologie (FMPOS), Bamako, Mali, and the US National Institute of Allergy and Infectious Diseases (NIH, Bethesda, MD, USA), as well as the Mali national regulatory authority and conducted under FDA IND 17130. Participants provided written informed consent to take part in the study. The clinical trial NCT02942277 was registered in clinicaltrials.gov and will be published elsewhere.

### Antigen formulation and immunizations

NCT02942277 was the first-in-human trial of Pfs25-EPA and Pfs230D1-EPA with AS01 to determine safety, immunogenicity, and functional activity of these vaccines in Malian adults. The PpPfs25 and EcEPA lots as well as the Pfs25-EPA conjugated nanoparticle vaccine were manufactured at Walter Reed Bioproduction facility (Silver Spring, Maryland) in cGMP compliance. PpPfs25 is a Pichia- expressed recombinant Pfs25 with a molecular mass of 18,713 Daltons. The Pfs25-EPA vaccine was vialed as conjugated Pfs25 in 4 mM PBS to a 2X dilution of the high dose (188 µg/ml in 0.5 ml volume) in cGMP compliance at Walter Reed Bio-production facility in April 2016 and provided as a single use vial. AS01B adjuvant was provided as a single use vial by GSK, containing 100 µg/ml MPL and 100 µg/ml QS21 in a liposomal formulation in a volume of 0.625 ml. One dose injected of AS01 in this study corresponded to 50 µg QS21 and 50 µg MPL.

The study first involved a dose escalating, comparator-controlled pilot safety adult cohort (*N* = 65) in a periurban community in Mali to evaluate the safety and immunogenicity of Pfs25 (16 µg, *N* = 5; 47 µg, *N* = 10), Pfs230D1 (13 vg, *N* = 5; 40 µg, *N* = 10), or combination of Pfs25 and Pfs230D1 (16 µg + 13 µg, *N* = 5; 47 µg + 40 µg, *N* = 10) versus comparator (Engerix-B, *N* = 20) administered at 0, 1, 6 months. Pfs25 hmAbs used in this study were generated from four subjects in the open label safety cohorts who received 47 µg Pfs25-EPA/AS01 (Supplementary Table 1). The subjects enrolled in the clinical trial were healthy nonpregnant adults aged 18–50 years with lifelong exposure to seasonal *P. falciparum* infection. PBMCs used in this study were collected seven days after the third immunization.

### Isolation and sequencing of Pfs25 mAbs

Heavy and light chain variable domains were determined by sequencing of Pfs25-specific single B cells^55^. These cells were sorted using flow cytometry after the enrichment with Pfs25 biotinylated probes. For that, human PBMCs from Malian adults were stained with CD3- (UCHT1), CD14- (M5E2), CD56- (HCD56) Alexa Fluor 700–, CD19 APC-CY7– (HIB19), and CD20 PE-CY7–conjugated (2H7) mAbs purchased from BioLegend. A gating strategy was performed first for doublet discrimination, then singlet cells were selected for exclusion of non–B cells using CD3, CD14, and CD56 markers (Supplementary Fig. 7)^55^. Lymphocytes were gated for CD19 + CD20 + . Pfs25-specific B cells were gated using PE and excluding unspecific binding with the fluorochrome used in the decoy BSA tetramer (CF594). Analysis was performed in FACSAria II (BD Biosciences) with blue, red, and violet lasers. Pfs25-specific B cells were analyzed according to the fluorescence staining profile and sorted directly into a 96-well plate using FACS with a nozzle of 100 µM. Plates were then centrifuged at 1278 x g for 30 s and placed at −80 °C. Amplification of IG heavy variable (VH) and light variable (VL) domains from single sorted cells was performed by iRepertoire Inc. Briefly, RT-PCR1 was performed with nested, multiplex primers covering heavy, κ, and λ loci, and including partial Illumina adaptors. A total of 500 bp fragments corresponding to the VH and to the VL domains were amplified from each single B cell.

### SMFA

Gametocyte cultures of P. falciparum NF54 strain were maintained for 16–18 days with daily medium change. On the day of feed, gametocytemia was determined by microscopy, and the culture was adjusted to 0.15–0.2% stage V gametocytemia at 50% haematocrit. The gametocyte culture (200 ul) was mixed with test hmAb at indicated concentration (in 60 ul of 1xPBS), and then immediately fed to ~50 of 3–6 days old female *Anopheles stephensi* mosquitoes through a membrane-feeding apparatus. The mosquitoes were kept for eight days and dissected (*n* = 20 per group) to enumerate the oocysts in the midgut from mosquitoes with any eggs in their ovaries at the time of dissection^56^.

The % inhibition in oocyst density (%TRA), the 95% confidence interval (95% CI) and *p*-value from a single or multiple feeds were calculated using a zero-inflated negative binomial (ZINB) model as before^57^ using R (version 4.1.2, The R Foundation for Statistical Computing)^58^.

### Expression and purification of Pfs25, scFvs, and IgGs

The amino acid sequence for the Pfs25 construct used for structural and biophysical studies was obtained from UniProt (P13829) and consisted of residues K23-T193. Three N-linked glycosylation sites at residues 91, 143, and 165 within the construct were mutated from Asn to Gln (Supplementary Fig. 2). This construct was cloned into the pHLsec plasmid with a C-terminus hexa-histidine tag.

Single chain variable fragments (scFvs) were designed by fusing the VH region of each mAb to its paired VL region by a (GGGGS)_4_ linker. All scFv constructs were also cloned into the pHLsec plasmid with a C-terminus hexa-histidine tag. The fifteen hmAbs used for initial screening were expressed in an IgG1 backbone by LakePharma Inc. The five hmAbs used for further characterization were expressed as IgGs by cloning the VH and VL regions into pHLsec plasmids containing human IgG1 heavy, IgG1κ, or IgG1λ constant regions.

All constructs were transiently expressed in Expi293 cells following manufacturer protocol (Thermo Fisher Scientific, Waltham, MA). Light-chain and heavy-chain plasmids were co-transfected using a 1:1 ratio. All constructs were expressed as secreted protein and harvested four to five days after transfection. After centrifugation, Pfs25 and scFvs were purified by loading the supernatant on Ni-Sepharose Excel resin (Cytiva, Marlborough, MA) and washing with 10 column volumes (CV) of wash buffer (25 mM Tris pH 7.4, 300 mM NaCl, 30 mM imidazole). Recombinant protein was eluted with five CV of elution buffer (25 mM Tris pH 7.4, 300 mM NaCl, 150 mM imidazole) and concentrated using an Amicon Ultra Centrifugal filter with 10 kDa MWCO (Millipore Sigma, Burlington, MA). Concentrated eluate was purified by size exclusion chromatography using a Superdex 75 Increase 10/300 GL column (Cytiva, Marlborough, MA) equilibrated in 20 mM Tris pH 8.0, 100 mM NaCl.

IgGs were purified by diluting the supernatant 1:1 in IgG binding buffer (Thermo Fisher Scientific, Waltham, MA), loading on Protein A resin, washing with 10 CV of IgG binding buffer (Thermo Fisher Scientific, Waltham, MA), and eluting with 15 CV of IgG elution buffer. Eluate was neutralized using 100 μl of 1 M Tris pH 9.0 per ml of eluate and then concentrated using an Amicon Ultra Centrifugal filter with 50 kDa MWCO (Millipore Sigma, Burlington, MA). Eluate was further purified by size exclusion chromatography using a Superdex 200 Increase 10/300 GL column (Cytiva, Marlborough, MA) equilibrated with 20 mM Tris pH 8.0, 100 mM NaCl.

### BLI – Epitope binning

All BLI experiments were performed using the OctetRED 96 (Sartorius, Fremont, CA) at 25 °C. Pfs25 was biotinylated in vivo at a C-terminus Avi-Tag in Expi293 cells by co-transfection with BirA in a 9:1 ratio. The media contained biotin at a concentration of 100 μM. After purification, Pfs25 and all scFvs were buffer exchanged into HBS-EP+ buffer (Cytiva, Marlborough, MA) using Zeba Microspin Desalting columns with a 7 kDa MWCO (Thermo Fisher Scientific, Waltham, MA). Pfs25 was then diluted to 10 nM and all scFvs were diluted to 150 nM in HBS-EP+ buffer.

Biotinylated Pfs25 was loaded on streptavidin biosensors (Sartorius, Fremont, CA) for 300 s and a baseline measurement was obtained for 60 s. The loaded sensors were then dipped into wells containing the saturating scFv (150 nM) for 600 s. A second 60 s baseline was obtained, and the sensors were then dipped into wells containing the competing scFv (75 nM) for 300 s. AS01-08 and AS01-50 were observed to have high off-rates from the Pfs25-coated sensor during the baseline and were excluded from the experiment as primary scFvs but were still suitable for use as competing scFvs. Data were analyzed using Octet Data Analysis HT 12.0 Software (Sartorius, Fremont, CA) and normalized to the binding of the saturating antibody in the absence of competing antibody to Pfs25 and by using self-subtraction.

### BLI – Binding kinetics

Binding kinetics experiments were carried out using IgG diluted to 5 nM in HBS-EP+ buffer (Cytiva, Marlborough, MA) and Pfs25 that was buffer exchanged into HBS-EP+ buffer using Zeba Microspin Desalting columns (Thermo Fisher Scientific, Waltham, MA). Each IgG was loaded onto Anti-Human Fc Capture biosensors (Sartorius, Fremont, CA) for 300 s for AS01-04, 600 s for AS01-50, and 360 s for AS01-63, before a 60 s baseline was obtained. The sensor was then dipped into a two-fold dilution series of Pfs25 with a starting concentration of 75 nM for AS01-04, 600 nM for AS01-50, and 500 nM for AS01-63 and then dipped into a well containing buffer alone to measure dissociation. Both steps were 300 s long. Data were analyzed using Octet Data Analysis HT 12.0 Software (Sartorius, Fremont, CA) and globally fit to a 1:1 binding model. Three biological replicates with three technical replicates were performed for each IgG.

### Complex formation, crystallization and structure determination

To form each scFv complex, Pfs25 and each scFv was mixed in a 1:1 molar ratio and incubated on ice for 30 minutes. The mixture was concentrated using an Amicon Ultra Centrifugal filter with 10 kDa MWCO (Millipore Sigma, Burlington, MA) and injected into a Superdex 75 Increase 10/300 GL column (Cytiva, Marlborough, MA) equilibrated with 20 mM Tris pH 8.0, 100 mM NaCl. Fractions were pooled and concentrated to 10–20 mg/ml before use in crystallization experiments.

Each protein solution was mixed in a 1:1 ratio with mother liquor and used to set up crystallization experiments at 18 °C. Crystals of Pfs25 in complex with AS01-04 were grown in 0.2 M potassium thiocyanate and 23% PEG 3350 using hanging-drop vapor diffusion and cryoprotected in 30% MPD. Crystals of Pfs25 in complex with AS01-50 were grown in 0.1 M lithium chloride, 0.1 M HEPES pH 7.5, and 25% PEG 6000 using hanging-drop vapor diffusion and cryoprotected in 30% PEG 400 before flash freezing in liquid nitrogen. Crystals of Pfs25 in complex with AS01-63 were grown in 2.2 M ammonium sulfate and 0.1 M sodium citrate pH 5.4 using hanging drop vapor diffusion and cryoprotected in 30% glycerol.

X-ray diffraction data were collected at the GM/CA and SER-CAT beamlines at the Advanced Photon Source, Argonne National Laboratory. Data were processed and scaled using XDS^59^. Molecular replacement was performed in PHASER^60^ using Pfs25 (PDB ID: 6phb)^38^ and an antibody model generated by aBodyBuilder server as search models^61^. Initial models were built using PHENIX autobuild and iterations of model building and refinement were carried out using COOT^62^ and PHENIX^60^ refine.

### Reporting summary

Further information on research design is available in the Nature Research Reporting Summary linked to this article.

### Supplementary information

Supplementary material

REPORTING SUMMARY

## Data Availability

Atomic coordinates and structure factors have been deposited in the Protein Data Bank under the following accession numbers: 8EZK, Pfs25 in complex with AS01-04; 8EZL, Pfs25 in complex with AS01-50; 8EZM, Pfs25 in complex with AS01-63. All other data generated or analyzed during this study are included in this published article (and its supplementary information files).
