# Supplementary material for: A human antibody epitope map of the malaria vaccine antigen Pfs25
Source: NPJ Vaccines. 2023 Aug 4;8:108. doi: 10.1038/s41541-023-00712-z (PMC10403551; doi:10.1038/s41541-023-00712-z)
Supplement: Supplementary file 1 — Supplementary material [file 41541_2023_712_MOESM1_ESM.docx]

**Supplementary Information for**

**A human antibody epitope map of the malaria vaccine antigen Pfs25**

Niharika Shukla, Wai Kwan Tang, Camila H. Coelho, Carole A. Long, Sara A. Healy, Issaka Sagara, Kazutoyo Miura, Patrick E. Duffy, Niraj H. Tolia

*Corresponding author: [niraj.tolia@nih.gov](mailto:niraj.tolia@nih.gov)

**Contents:**

Supplementary Figures 1-7

Supplementary Tables 1-8

**Supplementary Figure 1:** **Initial screening of human anti-Pfs25 hmAbs**. Fifteen hmAbs were tested at 100 μg/mL by SMFA. The %TRA (circles) and the 95% confidence interval (error bars) are shown. * ,  p<0.05 and *** , p<0.001. The five hmAbs with the highest TRA% (separated from remaining hmAbs by a dashed vertical line) were selected for further study.

**
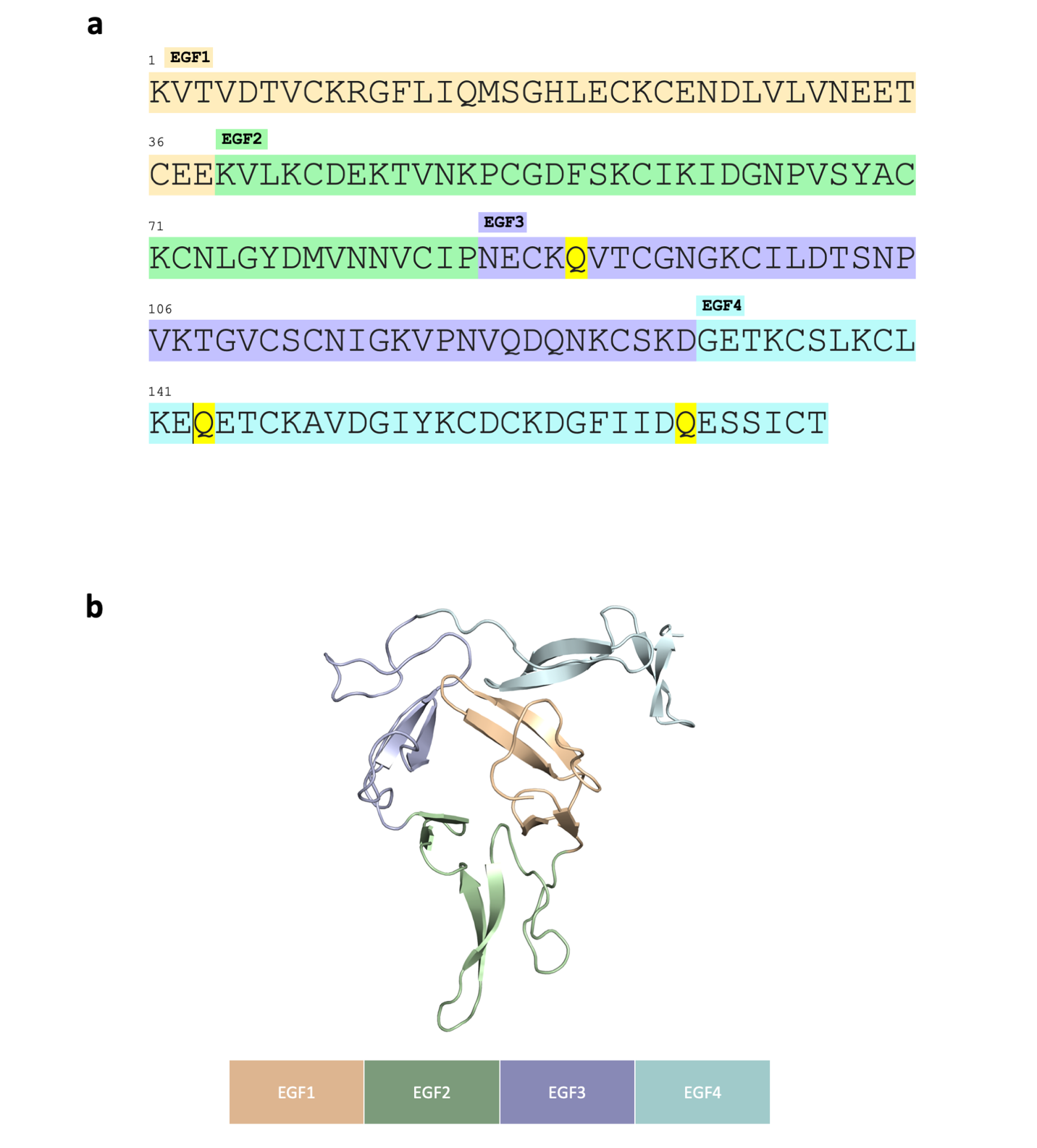
**

**Supplementary Figure 2: Pfs25 domain arrangement. a.** Primary amino acid sequence of Pfs25 construct used in epitope binning experiments. N-linked glycosylation sites are highlighted in yellow and were mutated to glutamine. **b.** Pfs25 depicted in cartoon representation with epidermal growth factor-like (EGF) domains 1-4 colored in orange, green, purple, and cyan.


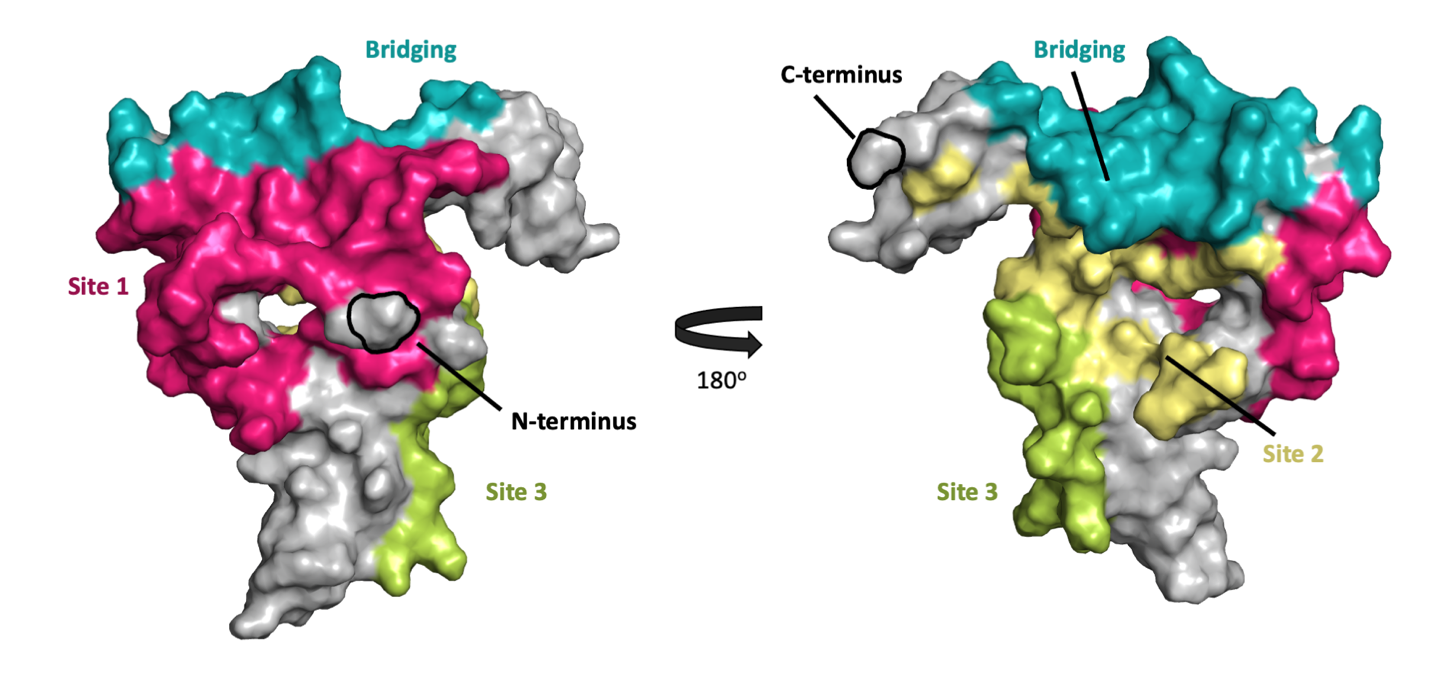


**Supplementary Figure 3:** **Schematic of previously identified immunogenic sites of Pfs25.** Pfs25 is depicted as a grey surface representation with N and C-termini outlined and immunogenic sites 1, 2, 3, and bridging epitope delineated by color^38, 39^.


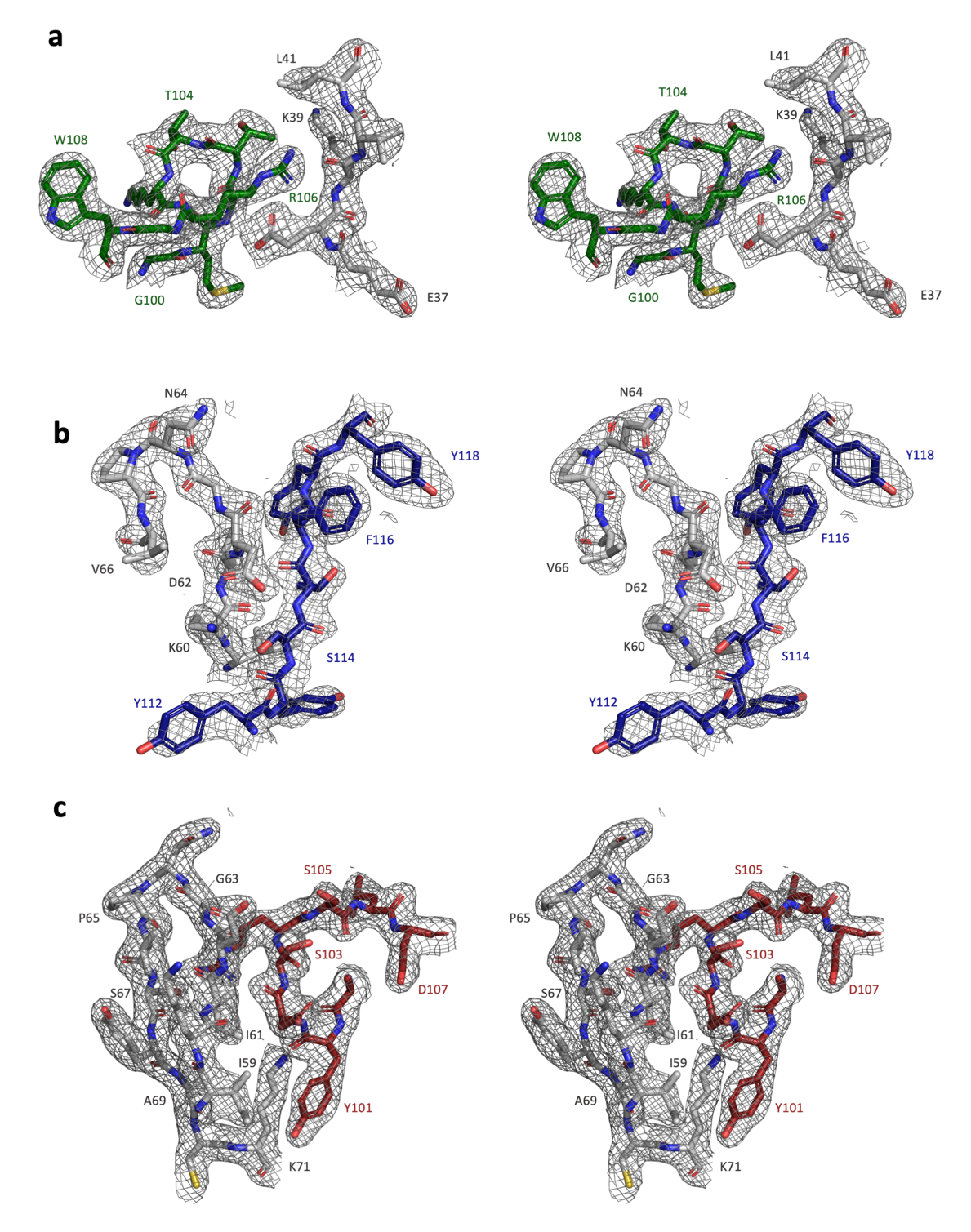


**Supplementary Figure 4: Stereo view of Pfs25 and hmAb interacting residues.** Pfs25 shown in grey and mAbs shown in green, blue, and red. **a.** AS01-04 HCDR3 and Pfs25. **b.** AS01-50 HCDR3 and Pfs25 **c.** AS01-63 HCDR3 and Pfs25. 2FO-FC electron density map contoured at 1σ.

**
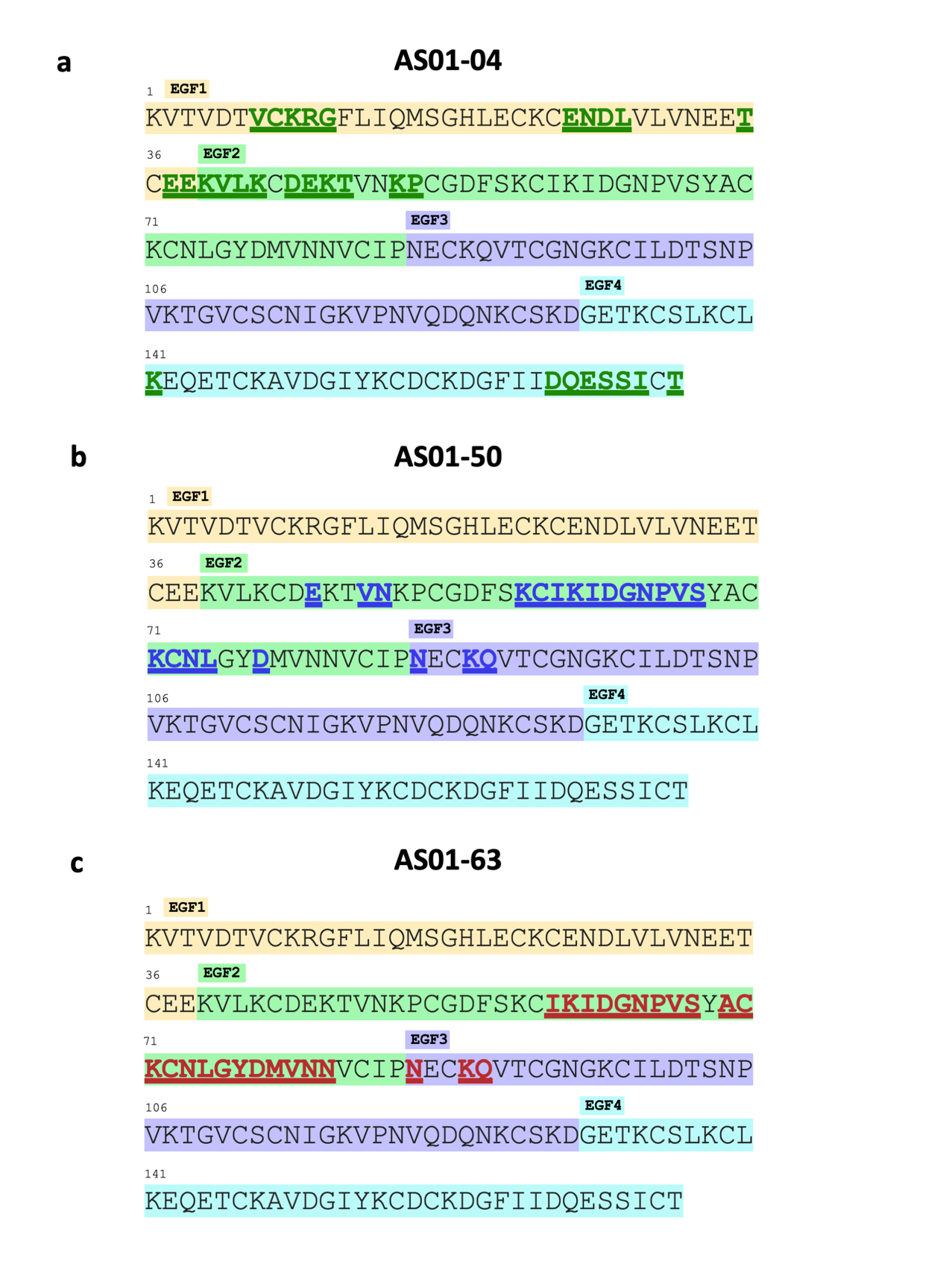
**

**Supplementary Figure 5: Amino acid sequences of Pfs25 hmAb epitopes.** Primary amino acid sequence of Pfs25 colored by EGF domain. Amino acids within epitopes of **a.** AS01-04, **b.** AS01-50, and **c.** AS01-63 are underlined and colored.


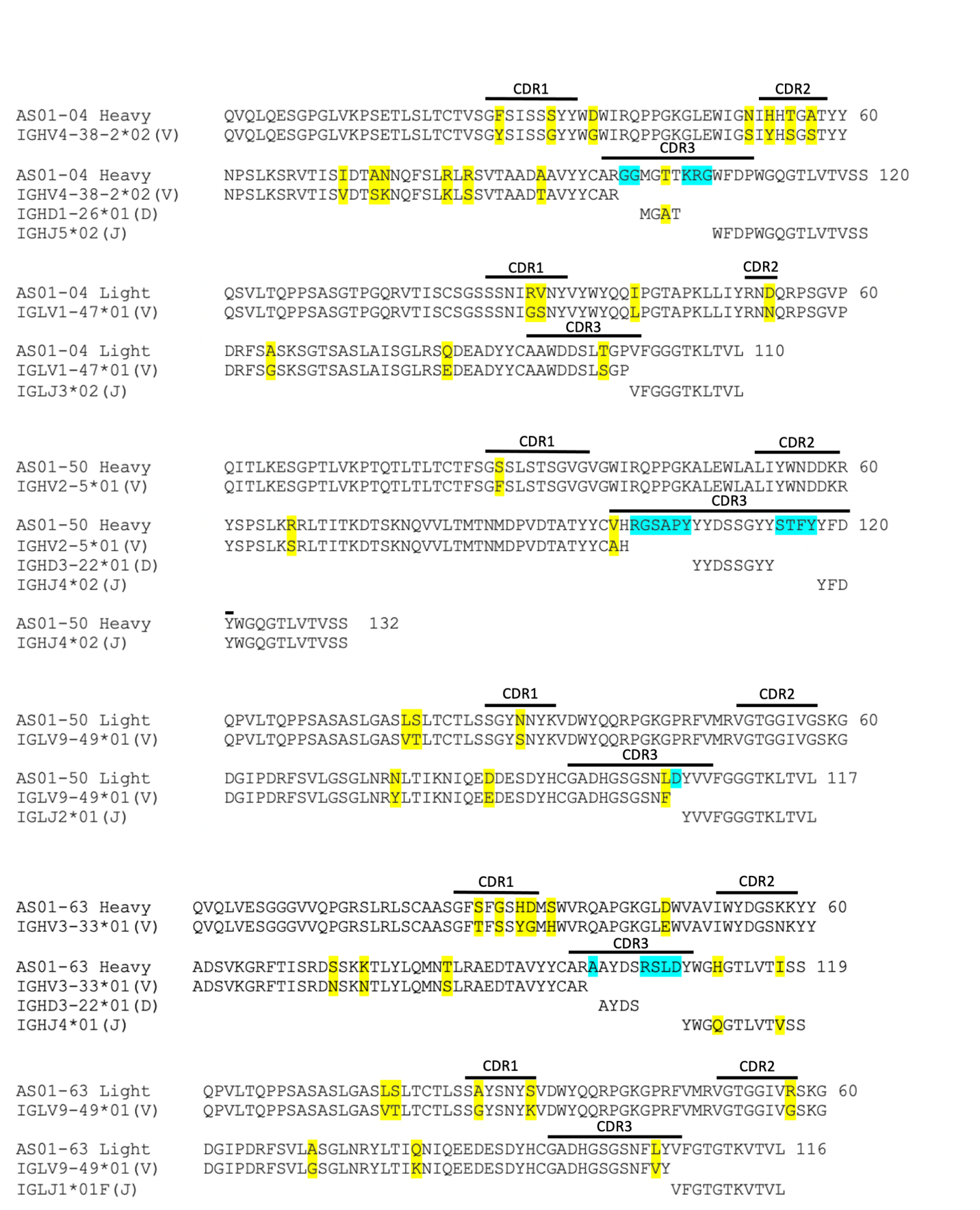


**Supplementary Figure 6: Germline analysis of Pfs25 hmAbs.** Comparison of antibody sequences to germline sequences, amino acids changes highlighted in yellow and insertions highlighted in cyan. CDRs as defined by IgBlast designated above antibody sequences.

**Supplementary Figure 7: Flow cytometry gating strategy to select for Pfs25-specific B-cells.** The bottom right panel identifies the antigen-specific single B cells from which VH and VL domains were amplified to generate the hmAbs used for initial screening in Suppl. Figure 1.

| **Subject** | **TRA**  **(day 112 -**  **3 months post dose 2)** | **TRA**  **(purified IgG, day 182 –**  **2 weeks post dose 3)** | **hmAbs isolated**  **(day 175 –**  **1 week post dose 3)** | |
| --- | --- | --- | --- | --- |
| **A** | 43.2% | 38.5% | AS01-02  AS01-03  **AS01-04**  AS01-06  AS01-07  **AS01-08**  AS01-17  AS01-19  AS01-25  **AS01-40**  AS01-41 |  |
| **B** | 54.5% | 24.2% | AS01-49  **AS01-50** |  |
| **C** | 56.9% | 54.3% | **AS01-63** |  |
| **D** | 42.9% | 49.1% | AS01-87 |  |

**Supplementary Table 1: Human mAb isolation.** The fifteen human mAbs used for initial screening were isolated from four Malian adults after immunization with Pfs25-EPA. The five human mAbs that were further characterized are indicated in bold. TRA% is indicated for each individual at 3 months post dose 2 (day 112) and 2 weeks post dose 3 (day 182).

|  | **Pfs25 in complex with AS01-04** | **Pfs25 in complex with AS01-50** | **Pfs25 in complex with AS01-63** |
| --- | --- | --- | --- |
| **PDB ID** | 8EZK | 8EZL | 8EZM |
| **Wavelength (Å)** | 1.00 | 1.03 | 1.00 |
| **Resolution range** | 38.1  - 2.3  (2.4  - 2.3) | 46.8  - 2.3  (2.4  - 2.3) | 44.5  - 2.1  (2.175  - 2.1) |
| **Space group** | P 21 21 21 | C 1 2 1 | C 1 2 1 |
| **Unit cell** | 68.25 91.72 122.10 90.00 90.00 90.00 | 88.61 55.75 86.09 90.00 103.63 90.00 | 101.89 84.71 65.87 90.00 96.57 90.00 |
| **Total reflections** | 275,467 (17,376) | 60,714 (4,930) | 107,091 (9,019) |
| **Unique reflections** | 34,702 (3,380) | 18,026 (1,731) | 30,366 (2,543) |
| **Multiplicity** | 7.9 (5.1) | 3.4 (2.8) | 3.5 (3.5) |
| **Completeness (%)** | 99.75 (98.66) | 98.29 (94.75) | 93.21 (77.68) |
| **Mean I/sigma(I)** | 10.30 (1.23) | 14.13 (2.25) | 14.00 (1.61) |
| **Wilson B-factor** | 50.85 | 48.36 | 59.38 |
| **R-merge** | 0.11 (1.16) | 0.05 (0.42) | 0.04 (0.58) |
| **R-meas** | 0.12 (1.30) | 0.06 (0.52) | 0.05 (0.68) |
| **R-pim** | 0.04 (0.56) | 0.04 (0.30) | 0.02 (0.36) |
| **CC1/2** | 99.7 (69.4) | 99.8 (81.5) | 99.8 (78.3) |
| **CC*** | 99.9 (90.5) | 99.9 (94.8) | 100 (93.7) |
| **Reflections used in refinement** | 34,685 (3,375) | 18,023 (1,732) | 30,352 (2,541) |
| **Reflections used for R-free** | 1,998 (195) | 1,802 (174) | 2,010 (170) |
| **R-work/R-free** | 22.93/26.03 | 21.65/25.76 | 20.50/23.72 |
| **Number of non-hydrogen atoms** | 6,176 | 3,317 | 3,067 |
| **macromolecules** | 6,157 | 3,249 | 3,023 |
| **ligands** | 0 | 14 | 24 |
| **solvent** | 19 | 54 | 20 |
| **Protein residues** | 818 | 430 | 398 |
| **RMS(bonds)** | 0.004 | 0.002 | 0.003 |
| **RMS(angles)** | 0.81 | 0.52 | 0.55 |
| **Ramachandran favored (%)** | 96.03 | 97.17 | 97.19 |
| **Ramachandran allowed (%)** | 3.97 | 2.83 | 2.81 |
| **Ramachandran outliers (%)** | 0.00 | 0.00 | 0.00 |
| **Rotamer outliers (%)** | 0.00 | 0.00 | 0.29 |
| **Clashscore** | 2.96 | 0.93 | 0.50 |
| **Average B-factor** | 68.26 | 59.63 | 74.15 |
| **macromolecules** | 68.32 | 59.74 | 74.04 |
| **ligands** | - | 75.45 | 100.64 |
| **solvent** | 49.27 | 48.93 | 59.10 |

**Supplementary Table 2: Crystallographic data collection and refinement statistics.**

**
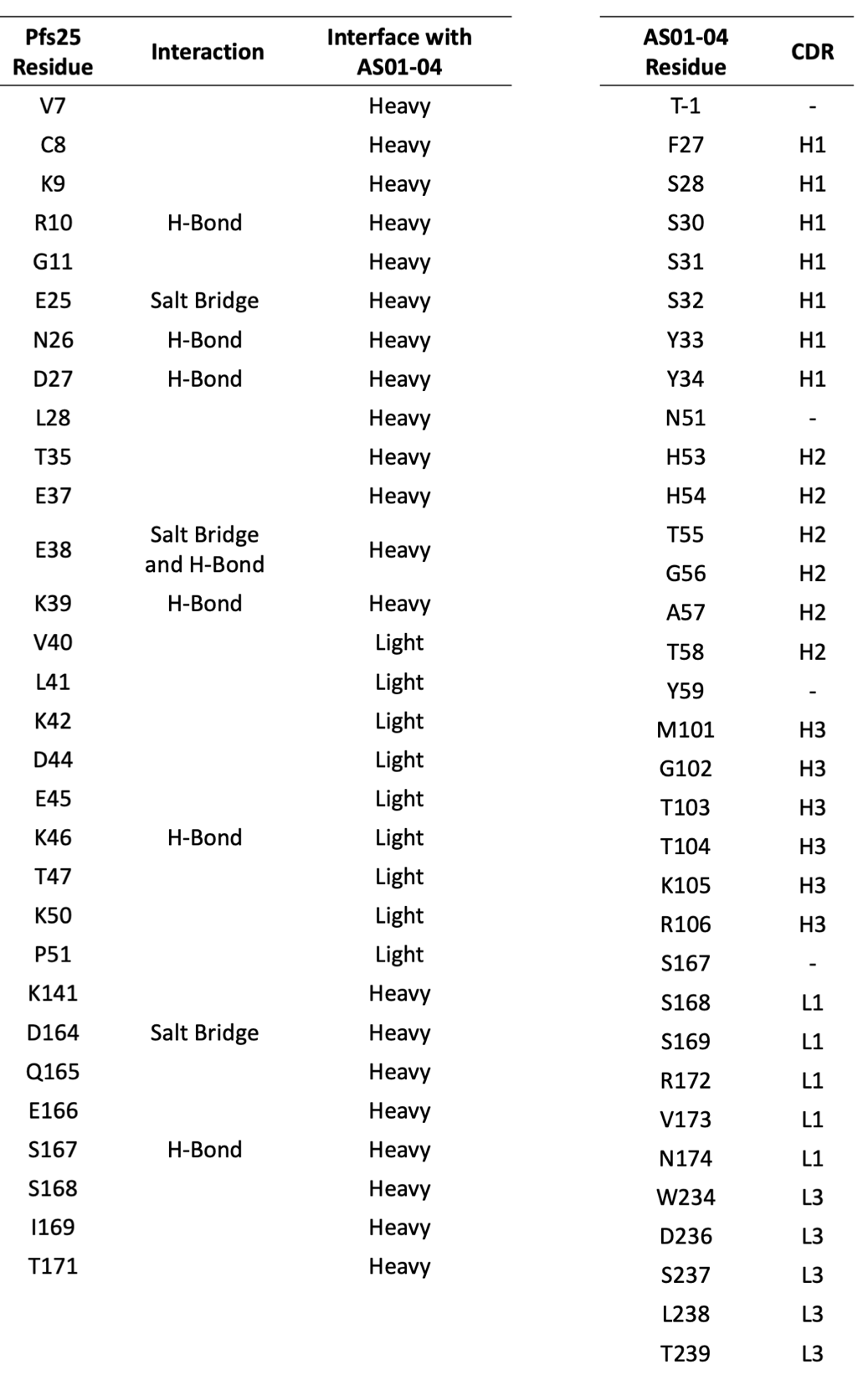
**

**Supplementary Table 3: Table of interacting residues for Pfs25 and AS01-04 scFv complex.** CDR indicates complementarity-determining region.

**
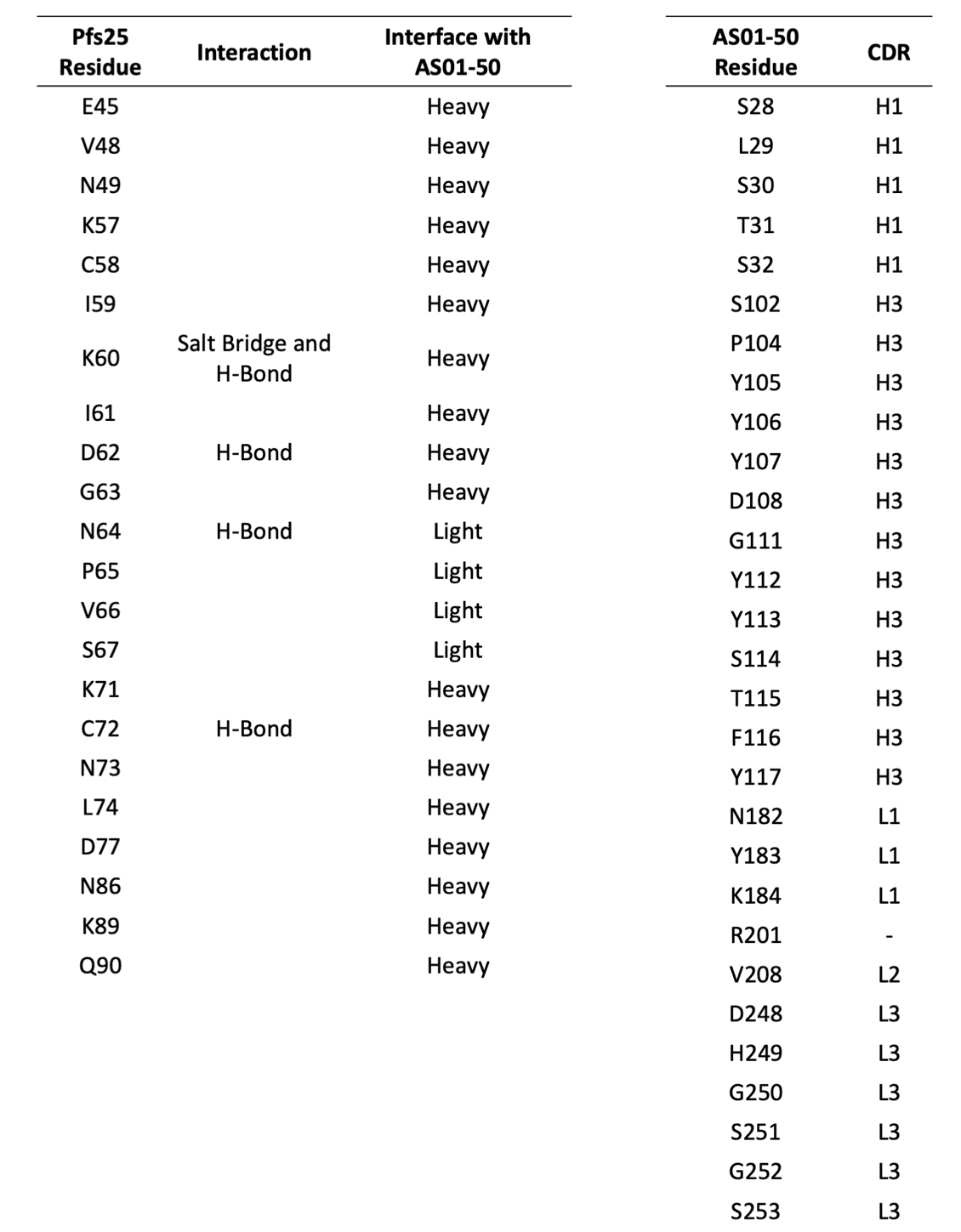
**

**Supplementary Table 4: Table of interacting residues for Pfs25 and AS01-50 scFv complex.** CDR indicates complementarity-determining region.


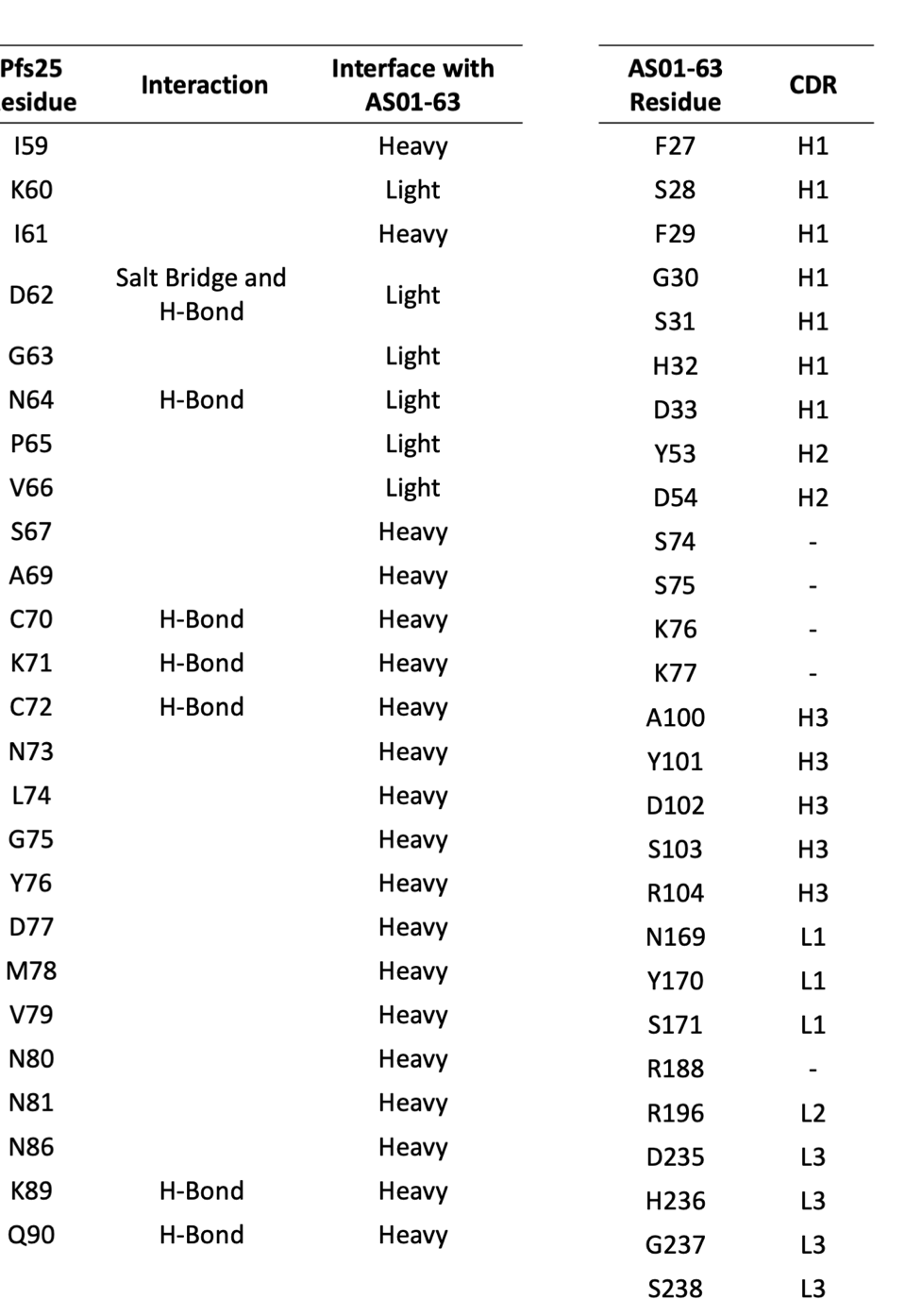


**Supplementary Table 5: Table of interacting residues for Pfs25 and AS01-63 scFv complex.** CDR indicates complementarity-determining region.

**
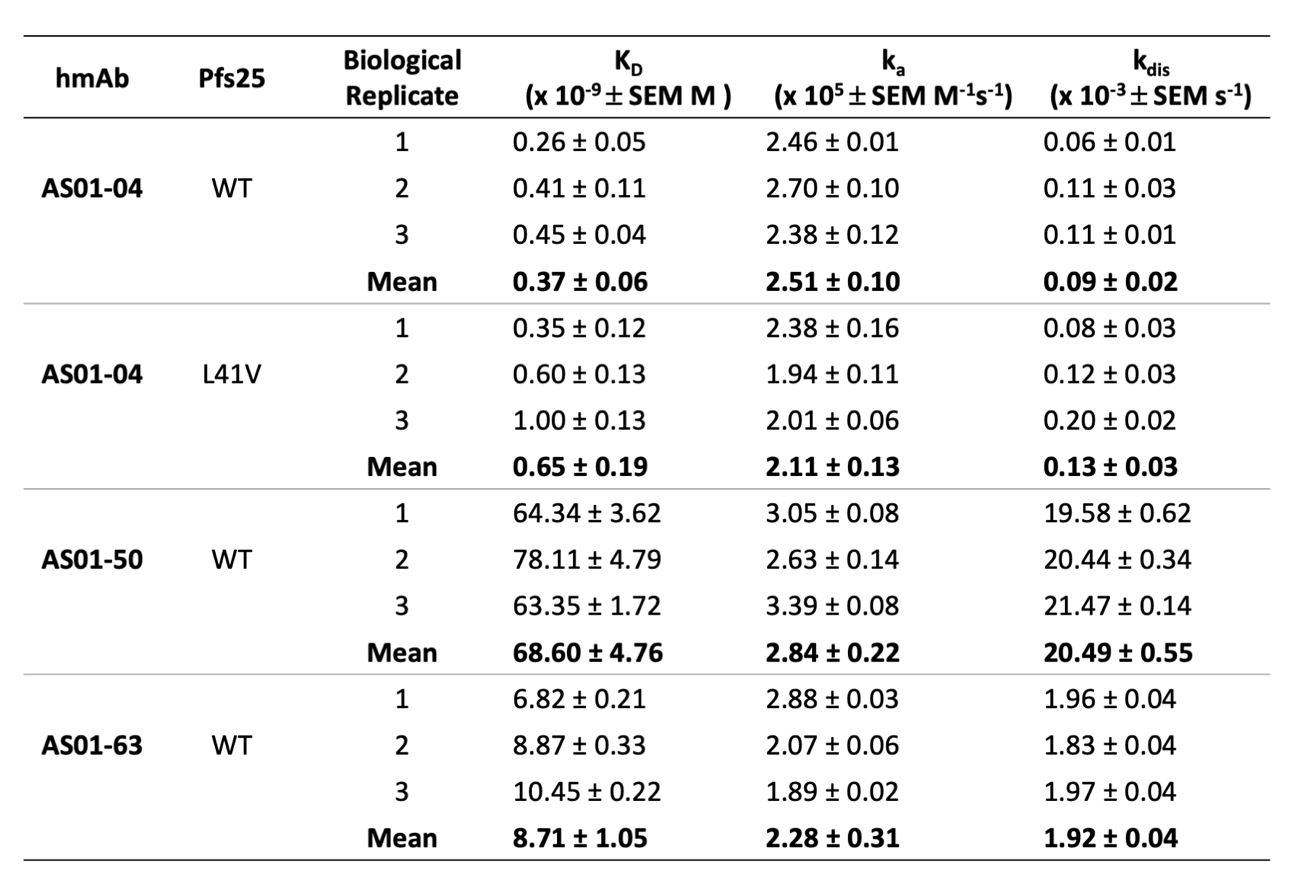
**

**Supplementary Table 6: Binding affinity and kinetics of AS01-04, AS01-50, and AS01-63 IgG.** Data for biological replicate indicates mean ± SEM from three technical replicates.


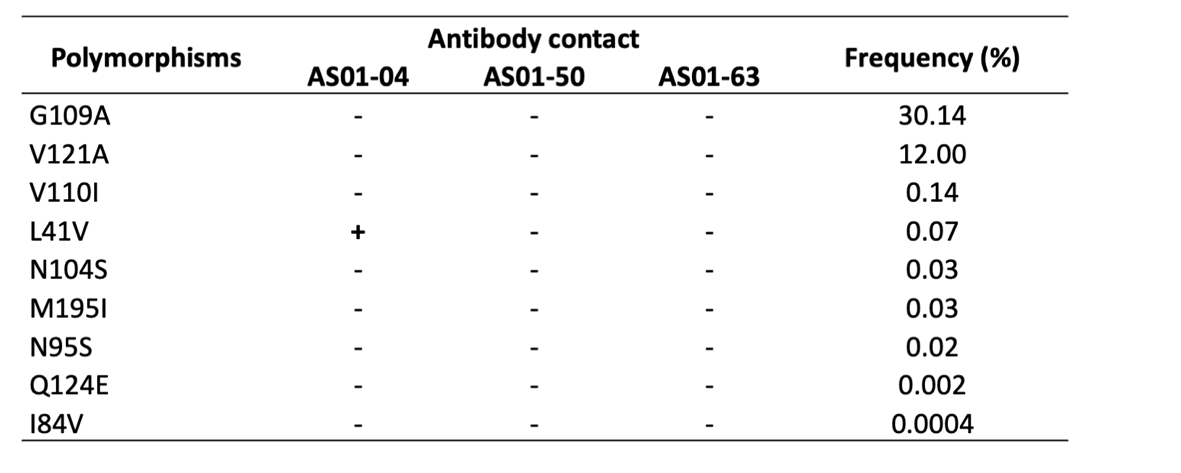


**Supplementary Table 7: Table of Pfs25 polymorphism frequency.** Frequencies derived from 3,488 sequences from MalariaGen. “+” indicates that polymorphic residue lies within epitope of mAb while “–” indicates that the residue lies outside the epitope.


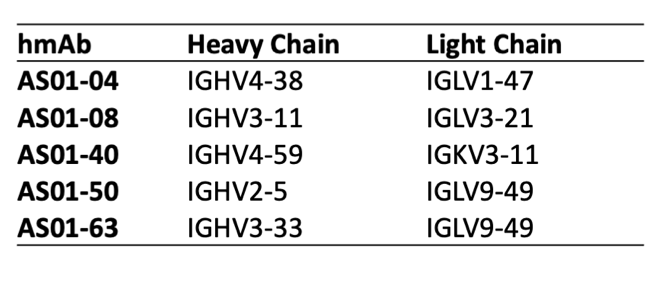


**Supplementary Table 8: Variable gene usage of Pfs25 mAbs.** Variable gene usage of heavy and light chains as defined by IMGT/VQuest.
