# Supplementary material for: A human antibody epitope map of the malaria vaccine antigen Pfs25
Source: NPJ Vaccines. 2023 Aug 4;8:108. doi: 10.1038/s41541-023-00712-z (PMC10403551; doi:10.1038/s41541-023-00712-z)
Supplement: Supplementary file 2 — REPORTING SUMMARY [file 41541_2023_712_MOESM2_ESM.pdf]

nature portfolio

Corresponding author(s): Niraj Tolia

Last updated by author(s): May 18, 2023

Reporting Summary

Nature Portfolio wishes to improve the reproducibility of the work that we publish. This form provides structure for consistency and transparency in reporting. For further information on Nature Portfolio policies, see our Editorial Policies and the Editorial Policy Checklist.

Statistics

For all statistical analyses, confirm that the following items are present in the figure legend, table legend, main text, or Methods section.

n/a	Confirmed	
☐	☒	The exact sample size (*n*) for each experimental group/condition, given as a discrete number and unit of measurement
☐	☒	A statement on whether measurements were taken from distinct samples or whether the same sample was measured repeatedly
☐	☒	The statistical test(s) used AND whether they are one- or two-sided<br>*Only common tests should be described solely by name; describe more complex techniques in the Methods section.*
☒	☐	A description of all covariates tested
☒	☐	A description of any assumptions or corrections, such as tests of normality and adjustment for multiple comparisons
☐	☒	A full description of the statistical parameters including central tendency (e.g. means) or other basic estimates (e.g. regression coefficient) AND variation (e.g. standard deviation) or associated estimates of uncertainty (e.g. confidence intervals)
☐	☒	For null hypothesis testing, the test statistic (e.g. *F*, *t*, *r*) with confidence intervals, effect sizes, degrees of freedom and *P* value noted<br>*Give P values as exact values whenever suitable.*
☒	☐	For Bayesian analysis, information on the choice of priors and Markov chain Monte Carlo settings
☒	☐	For hierarchical and complex designs, identification of the appropriate level for tests and full reporting of outcomes
☒	☐	Estimates of effect sizes (e.g. Cohen's *d*, Pearson's *r*), indicating how they were calculated

Our web collection on statistics for biologists contains articles on many of the points above.

Software and code

Policy information about availability of computer code

Data collection	UNICORN 7.3 software was used to collect FPLC data. FortéBio Data Acquisition 12.0 software was used to collect the bio-layer interferometry data. Gen5 3.08.01 software was used to collect the ELISA data. SERGUI and JBluIce was used to collect X-ray diffraction data.
Data analysis	- GraphPad Prism 9.2.0.<br>- FortéBio Data Analysis HT 12.0.<br>- XDS Package (version February 5, 2021), Phenix 1.16 and 1.20 (includes Phaser, AutoBuild, and Phenix.refine), Coot 0.9.8.1 and PyMol 2.5.1 curated by SBGrid.<br>- IMGT/V-QUEST online https://www.imgt.org/IMGTindex/V-QUEST.php.<br>- Clustal Omega online https://www.ebi.ac.uk/Tools/msa/clustalo/

For manuscripts utilizing custom algorithms or software that are central to the research but not yet described in published literature, software must be made available to editors and reviewers. We strongly encourage code deposition in a community repository (e.g. GitHub). See the Nature Portfolio guidelines for submitting code & software for further information.

Data

Policy information about availability of data

All manuscripts must include a data availability statement. This statement should provide the following information, where applicable:

- Accession codes, unique identifiers, or web links for publicly available datasets
- A description of any restrictions on data availability
- For clinical datasets or third party data, please ensure that the statement adheres to our policy

Atomic coordinates and structure factors have been deposited in the Protein Data Bank under the following accession numbers: 8EZK, Pfs25 in complex with AS01-04; 8EZL, Pfs25 in complex with AS01-50; 8EZM, Pfs25 in complex with AS01-63. All other data generated or analyzed during this study are included in this published article (and its supplementary information files).

Research involving human participants, their data, or biological material

Policy information about studies with human participants or human data. See also policy information about sex, gender (identity/presentation), and sexual orientation and race, ethnicity and racism.

Reporting on sex and gender	Fifteen monoclonal antibodies (hmAbs) were selected against Pfs25 from four Malian individuals vaccinated with Pfs25-EPA/AS01 regardless of sex and/or gender as sample selection was blinded to baseline characteristics and was based off positive interaction with Pfs25 from ELISA (n=15) and reduced further to five hmAbs with the greatest TRA.
Reporting on race, ethnicity, or other socially relevant groupings	Fifteen monoclonal antibodies (hmAbs) were selected against Pfs25 from four Malian individuals vaccinated with Pfs25-EPA/AS01 regardless of race, ethnicity, or other socially relevant groupings as sample selection was blinded to baseline characteristics and was based off positive interaction with Pfs25 from ELISA (n=15) and reduced further to five hmAbs with the greatest TRA.
Population characteristics	As described in Methods, "Antigen formulation and immunizations, lines 285-292" The study first involved a dose escalating, comparator-controlled pilot safety adult cohort (N=65) in a periurban community in Mali to evaluate the safety and immunogenicity of Pfs25 (16 µg, N=5; 47 µg, N=10), Pfs230D1 (13 vg, N=5; 40 µg, N=10), or combination of Pfs25 and Pfs230D1 (16 µg + 13 µg, N=5; 47 µg + 40 µg, N=10) versus comparator (Engerix-B, N=20) administered at 0, 1, 6 months. Pfs25 hmAbs used in this study were generated from four subjects in the open label safety cohorts who received 47 µg Pfs25-EPA/AS01 (Supplementary Table 1). The subjects enrolled in the clinical trial were healthy nonpregnant adults aged 18–50 years with lifelong exposure to seasonal P. falciparum infection.
Recruitment	Community permission was obtained from village elders and other community members in Sotuba/Bamako (periurban community) after explanation and discussion of the study at a community meeting. A general announcement inviting household and family members to the participating clinic to learn about the study was made at the time of community permission, using local radio or any traditional channel of communication.
Ethics oversight	As noted under Methods, "Human ethics statement, lines 265-268:" The trial protocol was approved by the ethics committees of the United States National Institute of Allergy and Infectious Diseases, National Institutes of Health; the Mali Faculté de Médecine de Pharmacie et d'OdontoStomatologie (FMPOS) of Bamako, and the Mali national regulatory authority, and conducted under FDA IND 17130.

Note that full information on the approval of the study protocol must also be provided in the manuscript.

Field-specific reporting

Please select the one below that is the best fit for your research. If you are not sure, read the appropriate sections before making your selection.

☒ Life sciences ☐ Behavioural & social sciences ☐ Ecological, evolutionary & environmental sciences

For a reference copy of the document with all sections, see nature.com/documents/nr-reporting-summary-flat.pdf

Life sciences study design

All studies must disclose on these points even when the disclosure is negative.

Sample size	Nine Pfs25-EPA/AS01, 47 µg subjects completed all three vaccine doses and four subjects were selected from the vaccine trial presenting higher antibody titers and functional activity for production of monoclonal antibodies.
Data exclusions	No data were excluded from this study.
Replication	Experiments were performed at least in duplicate. All attempts at replication were successful.
Randomization	In the clinical trial, Pfs25-EPA/AS01, 47 µg subjects were randomized, but not blinded, to receive high dose Pfs25 (47µg), Pfs230 (40µg) or comparator (Hepatitis B vaccine, Engerix-B), n=10/arm.
Blinding	In the clinical trial, Pfs25-EPA/AS01, 47 µg subjects were randomized, but not blinded, to receive high dose Pfs25 (47µg), Pfs230 (40µg) or

Blinding	comparator (Hepatitis B vaccine, Engerix-B), n=10/arm. The ELISA and SMFA assays performed on the serum samples were conducted in a blinded manner. Blinding of the investigators was not applied for other aspects of the study that examined functional and structural aspects of individual human mAbs.

Reporting for specific materials, systems and methods

We require information from authors about some types of materials, experimental systems and methods used in many studies. Here, indicate whether each material, system or method listed is relevant to your study. If you are not sure if a list item applies to your research, read the appropriate section before selecting a response.

Materials & experimental systems

n/a	Involved in the study
☐	☒ Antibodies
☐	☒ Eukaryotic cell lines
☒	☐ Palaeontology and archaeology
☒	☐ Animals and other organisms
☐	☒ Clinical data
☒	☐ Dual use research of concern
☒	☐ Plants

Methods

n/a	Involved in the study
☒	☐ ChIP-seq
☒	☐ Flow cytometry
☒	☐ MRI-based neuroimaging

Antibodies

Antibodies used	Heavy and light chain variable domains of the human antibodies were determined by sequencing of Pfs25-specific single B cells. Single chain variable fragments (scFvs) were designed by fusing the VH region of each mAb to its paired VL region by a (GGGGS)4 linker. All scFv constructs were also cloned into the pHLsec plasmid with a C-terminus hexa-histidine tag. The fifteen hmAbs used for initial screening were expressed in an IgG1 backbone by LakePharma Inc. The five hmAbs used for further characterization were expressed as IgGs by cloning the VH and VL regions into pHLsec plasmids containing human IgG1 heavy, IgG1K, or IgG1λ constant regions. All constructs were transiently expressed in Expi293 cells following manufacturer protocol (Thermo Fisher Scientific, Waltham, MA).
Validation	Antibodies were validated by sequencing, ELISA, and by measuring binding to Pfs25 by BLI (see manuscript)

Eukaryotic cell lines

Policy information about cell lines and Sex and Gender in Research

Cell line source(s)	Expi293F cells from ThermoFisher Scientific (cat# A14527)
Authentication	not authenticated
Mycoplasma contamination	not tested for mycoplasma
Commonly misidentified lines (See ICLAC register)	none

Clinical data

Policy information about clinical studies

All manuscripts should comply with the ICMJE guidelines for publication of clinical research and a completed CONSORT checklist must be included with all submissions.

Clinical trial registration	NCT02942277
Study protocol	Full protocol can be found via clinicaltrials.gov (https://clinicaltrials.gov/ProvidedDocs/77/NCT02942277/Prot_SAP_000.pdf)
Data collection	Study data was collected on paper CRFs and then put into a study specific DataFax electronic database. Data from CRFs were collected directly from subjects during study visits and telephone calls, or were abstracted from subjects' medical records. All essential documents listed in the ICH Good Clinical Practice Guideline are maintained in compliance with institutional, IRB, state, and federal medical records retention requirements, whichever is longest. Pilot safety cohort subjects who received Pfs25-EPA/AS01, 47 µg were recruited, screened, enrolled, vaccinated, and completed follow-up from December 2016 until August 2018, with last vaccinations completed in July 2017.
Outcomes	All relevant additional information as to safety and reactogenicity (primary), immunogenicity (by ELISA; secondary) and functional activity (by SMFA; secondary) of Pfs25, Pfs230 and combinations in Mali, including extensive analysis by ELISA and TRA, will be published in a separate manuscript and is beyond the scope of this study.
